# Inhibition of Personally-Relevant Angry Faces Moderates the Effect of Empathy on Interpersonal Functioning

**DOI:** 10.1371/journal.pone.0112990

**Published:** 2015-02-19

**Authors:** Vanessa Iacono, Mark A. Ellenbogen, Alexa L. Wilson, Philip Desormeau, Rami Nijjar

**Affiliations:** Centre for Research in Human Development, Concordia University, Montréal, Québec, Canada; University of Tuebingen Medical School, GERMANY

## Abstract

While empathy is typically assumed to promote effective social interactions, it can sometimes be detrimental when it is unrestrained and overgeneralized. The present study explored whether cognitive inhibition would moderate the effect of empathy on social functioning. Eighty healthy young adults underwent two assessments six months apart. Participants’ ability to suppress interference from distracting emotional stimuli was assessed using a Negative Affective Priming Task that included both generic and personally-relevant (i.e., participants’ intimate partners) facial expressions of emotion. The UCLA Life Stress Interview and Empathy Quotient were administered to measure interpersonal functioning and empathy respectively. Multilevel modeling demonstrated that higher empathy was associated with worse concurrent interpersonal outcomes for individuals who showed weak inhibition of the personally-relevant depictions of anger. The effect of empathy on social functioning might be dependent on individuals’ ability to suppress interference from meaningful emotional distractors in their environment.

## Introduction

Interpersonal relationships are essential in that they serve fundamental needs and contribute to overall health and well-being [1]. Interpersonal problems, such as difficulties being assertive, intimate, or sociable, are associated with maladaptive patterns of interpersonal functioning (e.g. social withdrawal) [2], higher mortality rates [3–5], and increased mental health concerns [6], [7]. Given the range of negative outcomes associated with interpersonal problems, understanding the causes and consequences of interpersonal dysfunction has become an important and timely research endeavor. In this regard, personality patterns have emerged as one key factor that might predispose certain people to experience problems when interacting with others [2], [8], [9]. Specifically, empathy is one such trait that has been closely linked to interpersonal functioning and has received considerable attention from investigators [10–12].

Empathy is defined as encompassing two qualitatively distinct yet interacting components. The first, affective sharing, describes an individual’s ability to vicariously experience an emotional response to another’s expressed emotions. The second, cognitive perspective-taking, refers to an individual’s capacity to adopt the subjective perspective of another [12–15]. While empathy is likely to vary as a function of a person’s current mood state [16], it is considered to be relatively stable across time and contexts, and to generalize across its affective and cognitive components [14].

The ability to communicate one’s own emotions and understand those of another is an inherent part of interpersonal functioning [17]. Because affective and cognitive aspects of empathy are linked with the accurate perception and understanding of emotional cues, it is often assumed that empathy will evolve into behaviors that promote effective social interactions [18]. In fact, the various benefits of empathic responding include enhanced well-being [19], self-esteem [20], and mental health [21], all of which have been implicated in the creation and maintenance of adaptive social relationships. While the majority of research links both cognitive and affective empathy to a wide range of relationship-enhancing effects, there is also evidence that empathy can be associated with adverse outcomes. For instance, recent studies have reported greater levels of empathy in aggressive children [22], adolescents with conduct disorder [23] and adults suffering from depression [24] relative to healthy controls, showing that high levels of empathy can occur in populations prone to experiencing poor interpersonal outcomes. The question remains as to what factors might influence the nature of empathy-related outcomes.

One hypothesis, initially put forth by Eisenberg and colleagues in the late 1980s [18], [25] (see for reviews), is that the effect of empathy on social functioning depends on one’s ability to regulate the vicarious experience of another’s emotion. In the absence of such control, excessive empathizing could lead to emotional overarousal, with the desire to alleviate one’s own discomfort taking precedence over the urge to attend to the other’s emotion. Where empathy evolves into self-focused rather than other-focused behaviors, the ability to perceive and understand another’s emotions will not necessarily promote effective social interactions [26–29]. Ultimately, because well-regulated individuals are believed to have control over the sharing of emotions between themselves and others, they may be better equipped to reach and maintain optimal levels of emotional arousal when empathizing with others [18], [28] Past research therefore emphasized the importance of self-regulation in modulating the emotional responses that ensued from empathizing with others [18]. Decety and colleagues [21], [30], [31] have recently expanded on the link between self-regulation and empathy within a more multifaceted framework of empathic understanding (i.e., including both affective and cognitive aspects of empathy), and emphasizing the role of executive functions (e.g., mental flexibility, planning, integration of information) and related effortful cognitive processes (e.g., attentional shifting).

**Fig 1:**
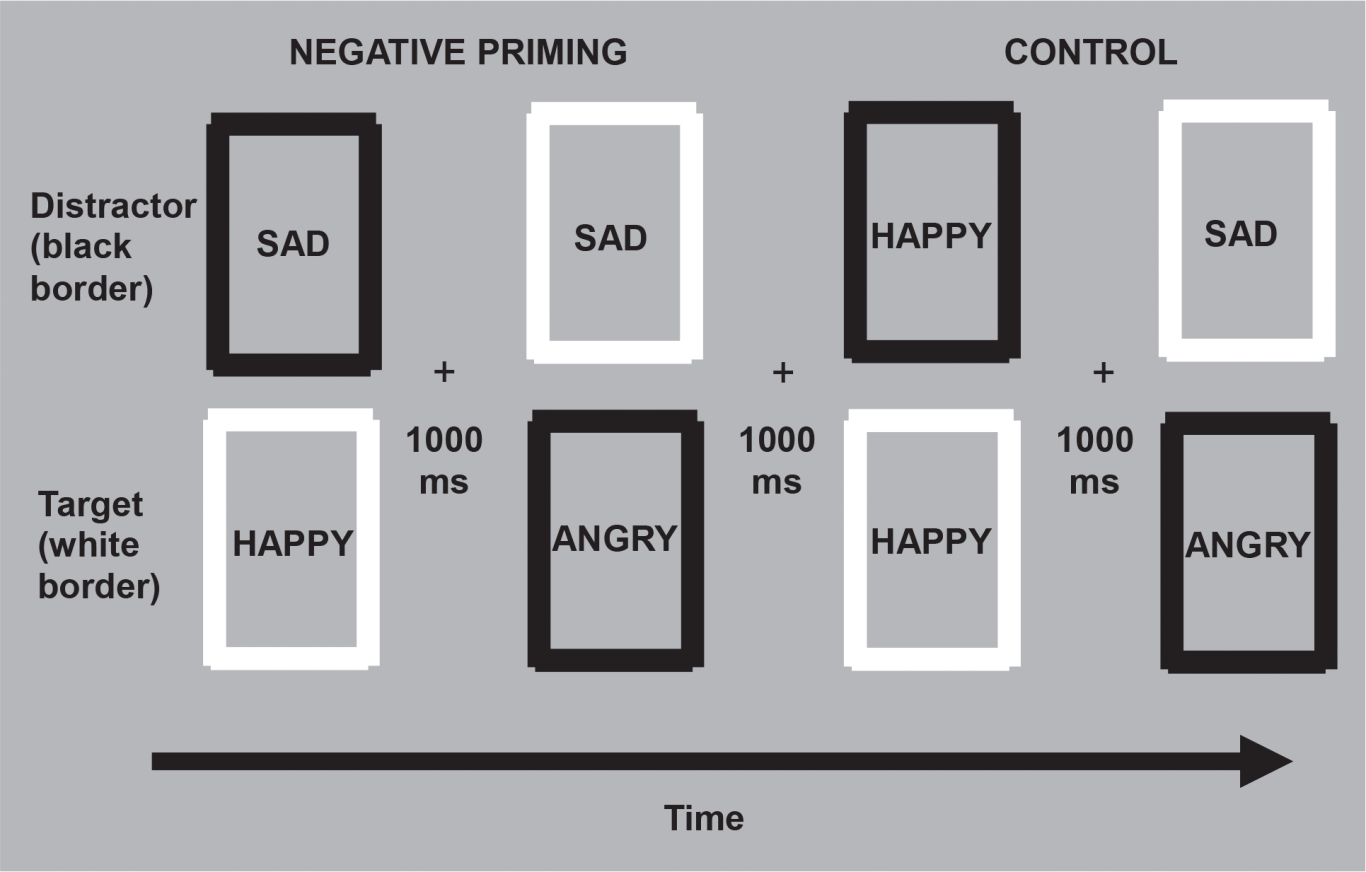
The NAP task was designed to assess participants’ ability to inhibit generic and personally-relevant facial stimuli depicting sad, happy, and angry emotional expressions. Target (white frame) and distractor (black frame) stimuli were presented simultaneously at either the top or the bottom of the screen and were preceded by the presentation of a centered fixation cross (1000 ms). Each paired trial consisted of a “test” presentation (columns 2 and 4) preceded by a “prime” presentation (columns 1 and 3). If the emotional expression of the distractor stimulus presented during the prime presentation (sad; top column 1) became the target emotional stimulus on the following test presentation (sad; top column 2), the trial was considered to be negatively primed. If both the target and distractor stimuli in the preceding prime presentation (happy; column 3) differed in emotional content from the target stimulus on the test presentation (sad; top column 4), the trial was regarded as a control. The design of the NAP task was identical for both the personally-relevant and generic stimuli. Note that publication restrictions prevent us from showing the actual visual stimuli used in this study.

Of particular interest to the present study is the role of cognitive inhibition, a component of executive functioning that regulates the content of working memory [32], [33] in modulating the effects of empathic responding during social interactions. Specifically, cognitive inhibition is defined as the ability to suppress interference from distracting or irrelevant information in the current environment [34]. Because working memory has a limited capacity, its efficient functioning depends on inhibitory processes that limit the access of irrelevant information into consciousness. When the capacity for cognitive inhibition is weakened, too much irrelevant information enters into working memory, hindering our ability to respond flexibly and adapt our behavior and emotional responses to the environment [32]. Individual differences in cognitive inhibition can be estimated with a negative priming paradigm [35]. In the modified version of the task, which assesses inhibition of emotional stimuli (i.e., faces of anger, sadness, and happiness) [36], [37] participants are instructed to respond to an emotional target stimulus while simultaneously ignoring or inhibiting an emotional task-irrelevant distractor. The negative priming effect refers to the delay in response latency that occurs when the emotional expression of the distractor stimulus that was inhibited on a previous trial becomes the target emotional expression on the subsequent trial (see Fig. 1). Ultimately, this delay is believed to represent the extent to which individuals showed strong suppression of interference from the emotional distractor during an ongoing, conscious activity. Conversely, speeded responding indicates facilitation, or that individuals showed poor/weak inhibition of the task-irrelevant emotional distractor [33].

Certain stimuli are harder for individuals to inhibit than others [38–41]. Depictions of emotional faces are a notable example of a salient biological and visual stimulus that tends to attract attention in an automatic, largely unconscious manner [42–45]. Few studies, however, have compared the ways individuals attend to generic and personally-relevant (meaningful and relevant to the participants) information, which are likely to be processed differently. In fact, there are neurobiological differences in the processing of familiar and unfamiliar faces. For instance, familiar faces elicit greater neural responses in the regions implicated in emotional processing, such as the amygdala and insula, compared to unfamiliar faces [46–50]. Despite mounting evidence to suggest that stimuli that are meaningful and relevant to individuals are processed differently, and perhaps more *readily*, than generic stimuli, the vast majority of work on emotional information processing has been limited to generic facial expressions of emotions as selected from validated databases.

Of primary interest to the present study was to examine whether cognitive inhibition moderates the relation between empathy and social functioning. Specifically, we investigated whether reduced inhibition of distracting emotional stimuli during a negative affective priming task could explain why some individuals with elevated levels of empathy experience negative interpersonal outcomes. Healthy young adults completed a negative affective priming task and were assessed at two time points, six months apart. The current study also expanded on previous research by exploring inhibition to pictures of both generic and personally-relevant (i.e. pictures taken from participant’s intimate partner) facial expressions of emotions (i.e. angry, sad, and happy). Of principal concern was whether emotional faces conveyed by actors who were meaningful and relevant to the participants would elicit pronounced inhibitory effects on a negative affective priming task, as this would support the use of personally-relevant stimuli in future studies of cognitive inhibition.

Two predictions were put forth. First, it was hypothesized that elevated levels of empathy would be associated with better interpersonal outcomes, but only for participants who showed strong inhibition of the distracting emotional stimuli. For those who showed poor inhibition of the emotional distractors, high empathy would be inversely related to social functioning. We examined these patterns at baseline, using concurrent measures, and prospectively by examining the relation between empathy and inhibition on interpersonal functioning six months later. Second, it was hypothesized that individuals would demonstrate greater response latencies to the personally-relevant expressions of anger compared to generic and sad and happy pictures, respectively. For individuals high in empathy, the meaningful and interpersonal nature of the personally-relevant expressions of anger might be especially challenging to inhibit given their heightened inclination to attend to emotional cues during social encounters [44]. Moreover, information processing on interpersonal themes may be more relevant to the prediction of social outcomes than the use of generic emotional stimuli, due to the strong links between biased processing of interpersonal information and social dysfunction as seen in various mental disorders [42], [51], [52]. Accordingly, we expected the moderation effect described in the previous hypothesis would be more pronounced for distractors from which interference was especially difficult to suppress.

Although we had no specific hypotheses, gender differences in empathy and cognitive inhibition, and their influence on social functioning, were assessed in the study. To test for the specificity of the relation between empathy and interpersonal functioning, secondary analyses using trait emotionality, agreeableness, and emotional intelligence as predictors of interpersonal functioning were examined. In addition, analyses using the quality of participants’ functioning in non-interpersonal domains (e.g., work, academic, health, finances) as the outcome were conducted for comparison.

## Method

### Participants

Healthy young adults between 18 and 32 years of age (*M* = 23.35, *SD* = 3.63) were recruited through advertisement in web-based services (i.e. Craigslist.com) and local community newspapers in the region of Montreal, Canada. For inclusion, participants were required to be in a romantic relationship of six months or longer. All participants were administered the Structured Clinical Interview for DSM-IV (SCID-I), Patient edition [53] by experienced doctoral-level clinical psychologists. Any past or present diagnosis of psychosis, schizophrenia, bipolar disorder, pervasive developmental disorder, or current substance abuse/dependence warranted exclusion from participation. Individuals were also excluded from participation if they had a visual impairment, major medical illness within the past three weeks, or were using psychotropic medication at the time of the study. Out of the 82 individuals initially contacted for this study, three had to be excluded because they met criteria for current substance/alcohol abuse. In the remaining study sample, eleven participants (14%) met criteria for either a current anxiety or mood disorder at the time of testing.

Assessments were made at two time points, six months apart. The initial sample consisted of 79 healthy young adults (50 females) and their intimate partners. Sixty-seven participants returned for assessment six months later, consisting of 85% of the original sample. The majority of participants reported being in a heterosexual relationship (94%), with approximately 50% being in their relationship for two years or more. Six (9%) of the returning participants had separated from their intimate partners in the interim between the first and second measurement occasions. No differences were observed between the original sample and those who dropped out six months later with regards to age, relationship length, lifetime diagnosis, neuroticism, empathy, inhibition, and social functioning (all *p* > .05). This study was conducted as part of a larger multidisciplinary longitudinal investigation of the biases in emotional information processing that underlie poor interpersonal functioning, and their relation to adrenal hormones and characteristics of the individual.

### Measures

Individual differences in trait cognitive and affective empathy were assessed through self-report using the short form of the Empathy Quotient (EQ-Short) [54]. Participants were asked to indicate the degree to which each of 22 statements accurately described them using a 4-point scale with anchors ranging from *strongly agree* to *strongly disagree*. In the current sample, the EQ-Short showed high internal consistency (α = .898), which is similar to what has been obtained in other studies using the same instrument [16], [54]. Studies support the EQ-Short as a valid measure of trait empathy [16] [54–56]. Neuroticism and agreeableness were measured using the Revised NEO Personality Inventory [57]. Emotional intelligence was assessed using the Mayer-Salovey-Caruso Emotional Intelligence Test (MSCEIT) [58], [59].

The chronic stress module of the UCLA Life Stress Interview [60] was used to evaluate participants’ functioning in four interpersonal (social life, close friendships, romantic relationships, and relationship with family members) and five non-interpersonal domains (education, work, finances, health of self, and health of family) over the previous six months. Each domain was coded on a five-point scale by the interviewer using behavior-specific anchor points and summed separately to create total interpersonal and non-interpersonal functioning scores. Higher scores reflect worse circumstances and social impairment. Interviewers were senior graduate students in clinical psychology that underwent extensive training on the instrument. Life domains comprising the interpersonal and non-interpersonal functioning composites showed moderate internal consistency (α = .686). Using independent interviewers' ratings of 20 participants, intra-class correlation coefficients revealed moderate to high inter-rater reliability for all domains, with a mean of 0.813.

### Personally-Relevant and Generic Stimuli

Using materials from the Facial Action Coding System (FACS) [61], the intimate partner of the participant was trained to generate three different facial expressions (angry, sad, and happy). Facial features particular to each emotional expression were demonstrated by the research assistant and described in detail (e.g., wrinkling of the nose, bearing of the teeth). Partners were also encouraged to use imagery to help evoke the required emotion and had a mirror at their disposal for practice. Approximately seven to ten pictures were taken of each facial expression using a digital camera mounted on a tripod. Five lab members then provided a global rating for each picture on a scale of 1 (*low*) to 10 (*high*) based on the degree of emotional intensity and genuineness conveyed by the facial expression. The two pictures with the highest average ratings for each emotion were included as stimuli in the study. The picture ratings for genuineness and intensity, averaged across all participants, for the angry, sad, and happy facial expressions, respectively, were as follows: M = 8.01, SD = 1.31; M = 8.05, SD = 1.43; M = 8.5, SD = .92. An intra-class correlation was computed and indicated high inter-rater reliability (ICC = .995) across the five lab members’ ratings of partners’ facial expressions.

The personally-relevant pictures were processed using Adobe Photoshop CS4 editing software. The pictures were reduced to a size of 170 by 231pixels and a color palette was applied to ensure that they were of the same brightness and hue as the generic pictures. With regards to the generic pictures, a total of 48 pictures were selected from the Karolinska Directed Emotional Faces database [62] in sets of 16 (8 male, 8 female actors, all Caucasian) angry, sad, and happy facial expressions.

### Negative affective priming (NAP) task

Derived from the original negative priming paradigm [35] and its recent adaptation [36], [63] a computerized cognitive task was designed to assess participants’ ability to inhibit generic and personally-relevant facial stimuli depicting sad, happy, and angry emotional expressions. Participants were instructed to use a two-key response box to identify whether the stimulus presented in the white frame (target) depicted a positive or negative facial expression, while ignoring the stimulus presented in the black frame (distractor). Target and distractor stimuli were presented simultaneously at either the top or the bottom of the screen and were preceded by the presentation of a centered fixation cross. Participants’ reaction time was recorded digitally (see Fig. 1).

Specifically, the NAP task consisted of fixed consecutive pairs of “prime” and “test” trials. Prime trials always preceded the test trials. In the negative priming condition, the emotional expression of the target picture during the test trial was the same as the emotional expression of the previously ignored distractor in the prime trial. In the control condition, the emotional expression of the target picture during the test trial was unrelated to the emotional expression of the previously ignored picture in the prime trial. Importantly, the pictures presented during the test trial of the negative priming and control conditions were identical; the conditions only differed in the pictures presented in the prime trials. Inhibition was assessed by measuring differences in reaction time between the negative priming test trials, where the emotion type of the target was previously ignored, and control test trials, where the emotion type of the target was unrelated to pictures in the previous trial. In order to counterbalance the type of emotional stimulus used as targets in prime and test trials, as well as distractors in test trials, two sequences of negative priming and control manipulations were used. Thus, half of the paired trials assessing the inhibition of each emotional category were designed according to the first sequence, and the other half were designed according to the second sequence. Trials were also counterbalanced for the spatial location of the pictures. The design of the NAP task was identical for both the personally- relevant and generic stimuli, and differed only in the number of distinct actors conveying the emotional expressions (i.e., the emotional expressions in the personally-relevant pictures were all conveyed by the participant’s intimate partner).

Given that the sole purpose of the prime trial was to vary the response to the test trial [64], only response times to the test trials were included in the statistical analyses. An index of inhibition was computed by subtracting mean reaction time on matched control test trials from mean reaction time on matched negative priming test trials. Calculations were performed separately for trials assessing inhibition to personally-relevant and generic pictures of angry, sad, and happy facial stimuli, yielding a total of six inhibition index scores. A positive index value indicates strong inhibition, meaning that the emotional expression of the distractor presented during the prime trial led to a slower reaction time during the test trial of the same emotion. Conversely, a negative index value indicates reduced/poor inhibition because the distractor presented during the prime trial prompted a faster reaction time during the test trial.

One hundred and ninety-two stimulus presentations were paired into 96 trials (48 negative priming trials and 48 control trials), which were viewed by each participant following a random sequence. Participants were presented with an equal number of paired trials for each emotional expression (32 sad, 32 happy, 32 angry), half of which consisted of personally-relevant pictures (16 personally-relevant and 16 generic sad, happy, angry faces respectively). Pictures remained on the screen until a response was provided or for a maximum of 7500 ms. Each trial was separated by an inter-stimulus interval of 1000 ms during which time a centered fixation cross would appear on the screen. The NAP task was run on an IBM-compatible computer with a with a 17-inch *NEC* color monitor. The STIM Stimulus Presentation software (version 7.584) created by the James Long Company (Caroga Lake, NY) was used to program the task as well as to record participants’ response times. The image resolution for the computer monitor was set to 800 x 600 pixels.

### Procedure

Following completion of the screening protocol, participants and their intimate partners were scheduled to come to the laboratory. Once signed informed consent had been provided, participants were administered the SCID-I/P and UCLA Life Stress Interview while their partner took part in a photography session in a separate room. Partners were instructed to wear a large grey t-shirt as to match the clothing in the personally-relevant pictures to that in the generic pictures. Once the photography session was completed, partners were debriefed and remunerated $20 CAN. The partners’ pictures would constitute the personally-relevant stimuli to which the participants would respond during the NAP task.

Approximately one week following their first appointment, participants were scheduled to return to the laboratory. Upon arrival, participants completed the EQ-Short, Revised NEO Personality Inventory, and MSCEIT. Following completion of the questionnaires, participants completed the NAP task described above. Participants were instructed to use a chin rest throughout the task to ensure that they remained seated at a distance of 57 cm away from the computer monitor.

Six months later, participants were contacted by telephone or e-mail and scheduled to return to the laboratory. Upon arrival, the quality of participants’ functioning during the six-month period since their last visit was assessed using the UCLA Life Stress Interview. One week following their first appointment, participants returned to the laboratory to complete the NAP task and were debriefed. Participants were remunerated $80 CAN per assessment for their participation in the study. All procedures were approved by the Human Research Ethics Committee of Concordia University (Montreal, Canada).

### Data Analysis

NAP reaction times below 300 ms and above 2000 ms as well as incorrect responses to test trials (5.5% of all reaction times) were excluded from the analyses. Skew (.305-.982) and kurtosis (-.550-.899) values for NAP reaction times were all within normal limits. Data were screened for outliers and distributional anomalies that may have violated statistical assumptions. Tests of bivariate and multicollinearity for all continuous predictors were also conducted and indicated no such incident in the current data set. Missing data for interpersonal functioning at the second measurement point (12.7%) were handled through multiple imputations using PASW version 19 [65].

To verify that significant negative priming occurred on the NAP task, a factorial analysis of variance (ANOVA) was initially performed on participants’ reaction time scores with Priming Condition (negative priming, control), Emotion Type (happy, sad, angry), Picture Relevance (pictures of intimate partner, pictures of strangers from a generic picture set), and Time (time 1, time 2) as within-subject factors. In these preliminary analyses, variables were entered simultaneously in a single GLM model and followed-up with planned comparisons for the main and interaction effects of interest. Next, given the non-independence inherent in longitudinal data, the main moderation analyses were conducted using a mixed effect model with maximum likelihood (ML) estimation using PASW version 19 [66]. In these analyses, individual data points for each participant were “nested” within time. An unstructured covariance structure was specified. While the time-dependent data served for within-subject comparisons (the level 1 units of analysis), the between-subject factors represented the level 2 units of analysis. In the within-subject analyses, participants’ total interpersonal functioning score at time 1 was used as the dependent variable and the timing of the data collection was used as the predictor. Linear effects of time were tested. In the between-subject analyses, individual differences in empathy and inhibition index scores for the generic and personally-relevant facial expressions of anger, sadness, and happiness were used to account for variability observed in the within-subject effects. Non-significant levels of between-subject variability in social functioning over time were followed-up with hierarchical multiple regression analyses that included a single data point as the dependent variable (i.e. predicting time 2 functioning).

Three separate multilevel models were analyzed for the personally-relevant and generic pictures of angry, sad, and happy faces respectively. Within each multilevel model, variables were entered hierarchically as follows. First, variables used as statistical controls (i.e., neuroticism and lifetime diagnosis) were entered into the model (step 1). Next, empathy, inhibition index scores for the generic and personally-relevant pictures of angry, happy or sad facial expressions (step 2), and their interactions (step 3) were entered into the model. A significant interaction was followed-up with a test of simple slopes [67]. Note that in these analyses, higher social functioning scores indicate greater impairment. Table 1 summarizes the data analysis plan and final results for the main multilevel analyses.

**Table 1:** Summary of linear mixed modeling results for predictors of the quality of participants’ social functioning.

			Effect			
			Fixed			Random
Variable			b	SE	t	Deviance
**Step 1** (intercept-only)						794.27**
Intercept			8.89*	.21	42.42	
**Step 2** (level 1)						3.77
Intercept			8.97*	.26	34.12	
Slope (linear Δ)			-.16	.25	-.628	
**Step 3** (level 2)^a^						60.53**
	**Model 1** ^b^ (Angry)					
		Intercept	6.18*	.77	8.05	
		Lifetime diagnosis	.002	.38	.01	
		Neuroticism	.03*	.01	3.83	
		EQ	.03	.02	1.29	
		PR	-3.01	1.71	-1.76	
		G	2.01	1.55	1.29	
		EQ X PR	-.56*	.19	-2.89	
		EQ X G	-.08	.16	-.49	
	**Model 2** (Sad)					60.68**
		Intercept	6.18*	.81	7.62	
		Lifetime diagnosis	-.08	.42	-.19	
		Neuroticism	.03*	.01	3.60	
		EQ	.02	.02	.76	
		PR	-.53	1.74	-.31	
		G	3.67	2.00	1.83	
		EQ X PR	-.14	.19	-.69	
		EQ X G	-.16	.26	-.61	
	**Model 3** (Happy)					55.27**
		Intercept	6.00*	.76	7.87	
		Lifetime diagnosis	.05	.39	.13	
		Neuroticism	.03*	.01	3.91	
		EQ	.02	.02	1.00	
		PR	3.05	1.70	1.93	
		G	-.13	1.79	-.07	
		EQ X PR	-.26	.20	-1.28	
		EQ X G	-.23	.23	-1.02	

*Note*. In these analyses, the intercept represented participants’ social functioning at time 1.

^a^ Slope was set as fixed at Step 3 due to non-significant change in model fit at Step 2. Predictors, added at Step 3, were used to explain between-subject variability in the intercept only.

^b^ Three models, distinguished by type of emotional stimulus, were run at Step 3. Within each model, variables were entered hierarchically (1- covariates, 2—main effects, 3—interaction effects).

EQ = empathy quotient; PR = personally-relevant; G = generic.

* p < .05

** Increase in model fit was statistically significant (*p* < .05) based on chi-square test of deviances.

## Results

### Preliminary analyses of the NAP task over time

To examine the impact of negative priming relative to control trials as well as the effects of personal-relevance and emotion type on participants’ reaction time on the NAP task across both measurement occasions, a Priming Condition (negative priming, control) X Emotion Type (angry, sad, happy) X Picture Relevance (personally-relevant, generic) X Time (time1, time2) repeated-measures ANOVA was conducted. Main effects for Priming Condition (*F*_(1,66)_ = 22.419, *p* < .001, *η* ^*2*^ *=* .007), Picture Relevance (*F*_(1,66)_ = 50.424, *p* < .001, *η* ^*2*^ *=* .026), Emotion Type (*F*_(2, 132)_ = 3.482, *p* = .034, *η* ^*2*^ *=* .003), and Time (*F*_(1,66)_ = 78.490, *p* < .001, *η* ^*2*^ *=* .024) were observed, with participants demonstrating significantly slower reaction times to negative priming rather than control test trials, personally-relevant compared to generic pictures, expressions of anger compared to happy and sad faces, as well as during the first compared to the second measurement occasion, respectively.

Importantly, the analyses yielded a significant Priming Condition X Picture Relevance X Emotion Type interaction (*F*_(2,132)_ = 3.480, *p* = .034, *η* ^*2*^ *=* .004). Follow-up tests of simple interactions were conducted using the per family error rate method (critical *F*-value = 3.79). These analyses indicated that the effects of emotion type on participants’ reaction time scores depended on the priming condition of the trial, but only for the personally-relevant (*F*_(2, 132)_ = 4.00, *p* < .05) compared to the generic (*F*_(2, 132)_ = 1.20, *p* > .05) pictures. Further probing using paired sample t-tests on participants’ reaction times to the personally-relevant pictures only, revealed significantly delayed response latencies to the negative priming compared to the control trials with regards to the facial expressions of anger (*t*_(78)_ = 4.387, *p* < .001, *d* = .99) and happiness (*t*_(78)_ = 2.14, *p* = .035, *d* = .49), demonstrating the expected negative priming effect. No significant differences between reaction times on negative priming and control trials were found for the sad faces (*t*_(78)_ = 1.063, *p* > .05, *d* = .24). Additional analyses found no evidence that the effects of Emotion Type and Picture Relevance on reaction time scores were moderated by gender (*p* > .05). Test-retest reliability coefficients, computed separately on raw reaction times for the faces of anger, sadness, and happiness on the negative priming and control trials, were .75, .78, 74, .80, .73, and .76 respectively for the personally-relevant stimuli, and .71, .81, .65, .74, .79, and 73 respectively for the generic stimuli, which was deemed sufficient for studying individual differences in inhibition [68]. Table 2 summarizes mean reaction times to negative priming and control test trials for the generic and personally-relevant pictures of anger, happiness, and sadness for the two measurement occasions.

**Table 2:** Response times (RTs) to negative priming and control test trials for generic and personally-relevant angry, sad and happy pictures in milliseconds at baseline and six months later.

		Generic Stimuli	Personally-Relevant Stimuli	Generic Stimuli	Personally-Relevant Stimuli
Time 1 (baseline)^a^					
	Sad Facial Stimuli	802 (147)	866 (147)	836 (156)	863 (172)
	Happy Facial Stimuli	823 (147)	845 (172)	799 (139)	819 (147)
	Angry Facial Stimuli	851 (188)	886 (180)	798 (156)	837 (164)
Time 2 (six months)^b^					
	Sad Facial Stimuli	717 (139)	785 (139)	742 (131)	751 (147)
	Happy Facial Stimuli	752 (106)	748 (139)	734 (115)	739 (131)
	Angry Facial Stimuli	724 (115)	773 (139)	711 (115)	745 (147)

*Note*. ^a^ *n* = 79

^b^ *n* = 67

Mean RTs (ms) are shown with standard deviations in parentheses

### Preliminary analyses of interpersonal functioning over time

**Fig 2:**
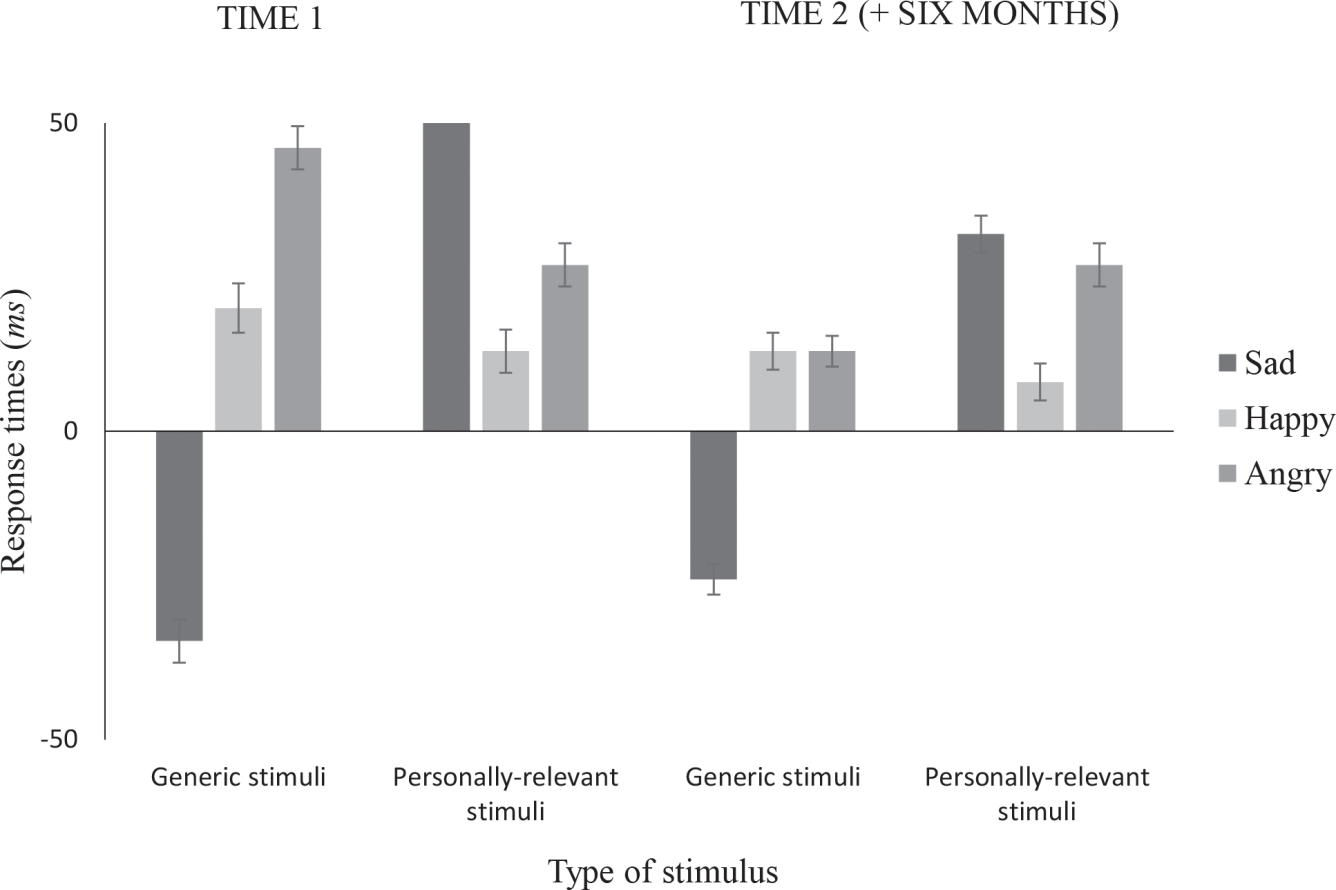
Mean inhibition index scores for sad, happy, and angry facial stimuli reported by measurement occasion in milliseconds. Index of inhibition scores were computed by subtracting mean reaction time on matched control test trials from mean reaction time on matched negative priming test trials. Errors bars represent standard errors.

All of the following analyses were conducted on inhibition index scores (i.e., negative priming minus control reaction times). Mean inhibition index scores and their standard deviations are presented by measurement occasion in Fig. 2.

A within-subject “unconditional” model was initially assessed that included only the dependent variable (i.e. interpersonal functioning at time 1; higher scores indicate worse social impairment). An intra-class correlation revealed that 43.14% of the total variability in interpersonal functioning at time 1 occurred between individuals, with the rest due to within-subject variability over the six months. Next, the time variable (level 1) was added as a linear predictor of change in interpersonal functioning across both time points. There was no significant linear change in interpersonal outcomes over time (*b* = -.162, *SE* = .284, *t*_(74)_ = -.569, *p* > .05), indicating that the quality of participants’ interpersonal functioning remained stable over the two measurement occasions.

### Predicting interpersonal functioning at time 1 and change over time

Next, between-subject effects (level 2) were examined. There was a significant amount of variability in the intercept for time 1 interpersonal functioning (χ^2^_(1)_ = 13.724, *p* < .001), meaning that participants differed in the quality of their interpersonal functioning at the beginning of the study. However, the between-subject variability in the linear effect of time (slope) was not statistically significant (χ^2^_(2)_ = 1.084, *p* > .05), indicating that between-subject effects did not influence the slope of interpersonal functioning across the two time points. Accordingly, the slope was set as fixed at level 2 such that the model made no attempt to explain variability in this effect. In other words, the remaining multilevel analyses pertained to the prediction of interpersonal functioning at time 1 (intercept).

Between-subject control variables including neuroticism and lifetime diagnosis were added as predictors of the variability in the time 1interpersonal functioning intercept. Only neuroticism had a statistically significant effect (*b* = .032, *SE* = .008, *t*_(75)_ = 3.859, *p* < .001), which explained 17.44% of the variability in time 1 interpersonal functioning. Then, empathy and indices of inhibition for the generic and personally-relevant facial expressions of anger were added to the model. There was no significant main effect of empathy (*b* = .028, *SE* = .022, *t*_(75)_ = 1.29, *p* > .05) or inhibition (*b* = -3.01, *SE* = 1.71, *t*_(75)_ = -1.76, *p* > .05) on the interpersonal functioning intercept. Next, the interaction between empathy and inhibition of generic and personally-relevant facial expressions of anger was included in the model. There was a significant empathy by inhibition of personally-relevant angry faces interaction (*b* = -.555, *SE* = .206, *t*_(75)_ = -2.691, *p* = .007), which explained an additional 8.38% of the between-subject variability on the interpersonal functioning intercept.

**Fig 3:**
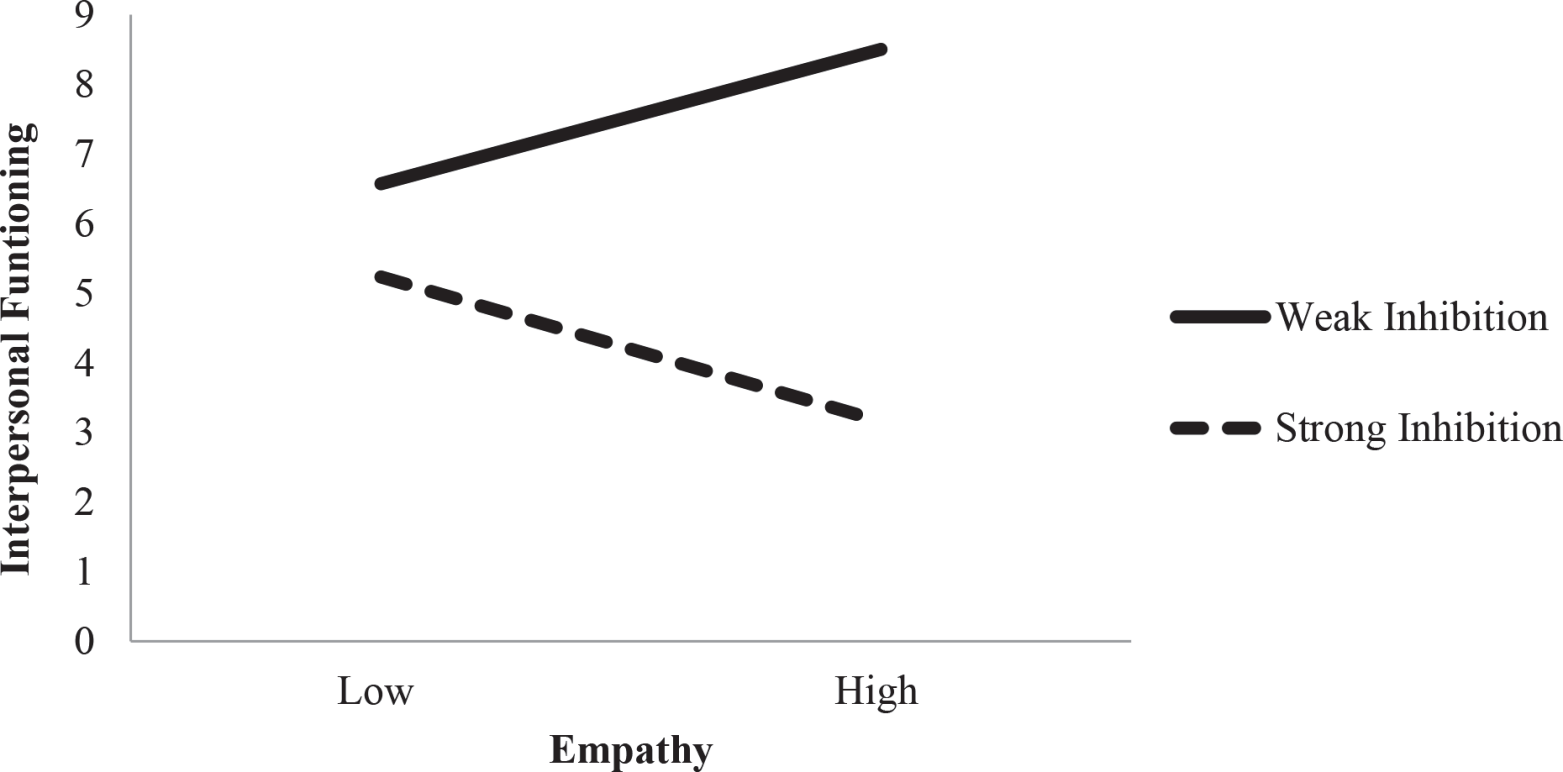
Simple slopes depicting the relation between empathy and interpersonal functioning at time 1 for individuals who inhibited (1 SD above the mean) the distracting personally-relevant facial expressions of anger and those who did not (1 SD below the mean). Note that higher interpersonal functioning scores indicate greater social impairment. For individuals who showed weak inhibition of the distracting angry face of their partner, elevated levels of empathy were associated with poor interpersonal outcomes (*b* = .057, *p* = .019). Conversely, for those who showed strong inhibition of the distracting angry face of their partner, high empathy was associated with positive interpersonal outcomes, but the slope was not statistically different from zero (*b* = -.061, *p* = .15).

Simple slopes analyses were conducted to examine the effect of empathy on interpersonal functioning at time 1 among participants who showed weak inhibition (1 SD below the mean) and those who showed strong inhibition (1 SD above the mean) of the distracting personally-relevant facial expressions of anger. For individuals who showed weak inhibition of the distracting angry faces of their partner, the slope depicted a positive relation between empathy and interpersonal functioning, which was significantly different from zero (*b* = .057, *t*_(71)_ = 2.393, *p* = .019, *pr* ^2^ = 3.14). That is, elevated levels of empathy were associated with poor interpersonal outcomes for individuals who demonstrated reduced suppression of interference from the personally-relevant depictions of anger (see Fig. 3). Conversely, for those who showed strong inhibition of the distracting angry faces of their partner, high empathy was related to positive interpersonal outcomes, but the slope was not statistically different from zero (*b* = -.061, *t*_(71)_ = -1.632, *p* = .15, *pr* ^2^ = .027). These findings suggest that cognitive inhibition becomes an important factor in predicting the effects of empathy on social functioning when individuals’ ability to suppress interference from salient emotional distractors in their environment is compromised.

The multilevel analyses described above were repeated with the indices of inhibition for the generic and personally-relevant facial expressions of happiness and sadness as moderator variables, and participants’ functioning in non-interpersonal domains (i.e. work, academic, health, finances) at time 1 as the outcome. None of these analyses yielded statistically significant results (all *p* > .05).

### Predicting interpersonal functioning at the second assessment

Because there was no evidence that inhibition and empathy predicted change in interpersonal functioning over time (see previous section), we could not use multilevel statistical techniques to examine between-subject variance in social functioning at the second time point. Rather, hierarchical multiple regression analyses were conducted in order to investigate whether empathy and inhibition at time 1 would predict interpersonal outcomes at the six-month follow-up. Similar to the previous analyses, the main effects for empathy (*b* = .019, *t*_(66)_ = .632, *p* = .530, *pr* ^2^ = .073) and inhibition (*b* = -2.592, *t*_(66)_ = -1.056, *p* = .295, *pr* ^2^ = -.122) of the distracting angry faces of their partner on prospective interpersonal functioning were non-significant. However, inhibition index scores for personally-relevant emotional distractors (*b* = -.354, *t*_(66)_ = -1.260, *p* = .212, *pr* ^2^ = -.145) did not moderate the effect of empathy on the quality of social functioning at the six-month follow-up. Thus, the interaction between inhibition and empathy predicted concurrent, but not prospective, interpersonal functioning. The regression analyses described above were repeated with the indices of inhibition for the generic and personally-relevant facial expressions of happiness and sadness as moderator variables (all *p* > .05). We also repeated the regression analyses excluding six participants who were no longer, at time 2, romantically involved with their partner from time 1. Neither empathy, inhibition index scores, nor their interaction significantly predicted interpersonal functioning at the second assessment (data not shown). Table 3 summarizes the main multiple regression results.

**Table 3:** Hierarchical multiple regression analyses using empathy and inhibition index scores by type of facial stimulus (measured at time 1) to prospectively predict the quality of participants’ social functioning at the six-month follow-up.

		Type of facial stimulus					
		Angry		Sad		Happy	
Predictor		Δ*R* ^*2*^	b	Δ*R* ^*2*^	b	Δ*R* ^*2*^	b
Step 1		.147		147		147	
	Neuroticism		.017		.014		.015
	Lifetime Diagnosis		1.11		1.31*		1.58*
Step 2		.019		.050		.156	
	EQ		.019		.014		.050
	PR		-2.60		4.84		2.81
	G		.587		.773		.210
Step 3		.036		.011		.042	
	EQ X PR		-.354		-.180		-.346
	EQ X G		.161		-.159		.337
Total R^2^		.202		.208		.345	

*Note*. Because predictor variables failed to account for *change* in participants’ social functioning scores over the two assessment periods (see Table 2 for multilevel analyses), hierarchical multiple regression was used to prospectively predict social functioning at the six-month follow-up from time 1 inhibition and empathy scores.

EQ = empathy quotient; PR = personally-relevant; G = generic.

* p < .05

### Supplementary analyses

Additional analyses were carried out using the neuroticism scale of the NEO-PI-R and managing emotions scale of the MSCEIT to examine whether the observed interaction effect was specific to empathy rather than general trait emotionality or emotional intelligence. The interaction between neuroticism and inhibition of generic and personally-relevant facial expressions of anger, sadness, and happiness, as well as their interaction with emotional intelligence, did not significantly predict interpersonal functioning at time 1 (all *p* > .05), suggesting that the present findings are specific to empathy. Supplemental analyses also found no evidence that the effect of empathy or inhibition index scores on interpersonal functioning was moderated by gender, nor was there a significant main effect of the agreeableness scale of the NEO-PI-R on social functioning at time 1when included as a covariate in the main multilevel analyses (all *p* > .05)

## Discussion

In the present study, we examined whether cognitive inhibition of emotional content could explain, in part, why elevated levels of empathy are associated with both positive and negative interpersonal outcomes. In contrast to studies using pictures from validated databases [36], [64], [69], the present study was among the first to include pictures of emotional facial expressions that were personally-relevant and meaningful to the participant, in addition to the use of generic pictures. Moreover, participants were evaluated at two time points, six months apart, to test the relation between empathy, cognitive inhibition, and social functioning using a prospective design. Two noteworthy results were found in this study. First, the use of personally relevant facial expressions of emotion, using pictures of participants’ intimate partners, elicited more inhibition than generic stimuli and were specifically related to interpersonal functioning; a relationship that was not detected with generic stimuli. Second, cognitive inhibition moderated the relation between empathy and the quality of interpersonal functioning. Among participants who showed weak inhibition of distracting personally-relevant facial expressions of anger, elevated levels of empathy were concurrently related to *adverse* social outcomes. Among those who showed strong inhibition of personally-relevant facial expressions of anger, high empathy was associated good interpersonal functioning, although this relation did not achieve statistical significance in follow-up multiple regression analyses for time 2.

The finding that empathy predicts poor interpersonal functioning when participants display weak inhibition of distracting personally-relevant facial expressions of anger is not only consistent with the proposal that some degree of self-regulation is required to optimize levels of empathy during interpersonal encounters [18], [21], [26], [30], [31], but also expands on previous research by demonstrating that high empathy can be maladaptive when there is insufficient inhibitory control over the processing of emotional information. Importantly, this finding was only observed for individuals who showed reduced inhibition of the personally-relevant facial expressions of anger. This suggests that differences in the quality of interpersonal functioning might only manifest when people are required to inhibit stimuli from which interference is especially difficult to suppress and, accordingly, more taxing on their cognitive resources. Stated otherwise, cognitive inhibition might only become a key factor in predicting the effects of empathy on social functioning when individuals’ ability to suppress interference from salient distractors in their environment is compromised.

In contrast, cognitive inhibition at time 1 did not moderate the relation between empathy and interpersonal functioning at the six-month follow-up. Thus, while the interaction between empathy and cognitive inhibition predicted concurrent social functioning, it failed to predict future interpersonal outcomes. One possibility is that factors unaccounted for between the two measurement occasions, including unmeasured variability in empathy, served to undermine the accurate prediction of social outcomes over time (i.e., decrease in prediction power over time). It is also likely that while participants’ general inhibitory abilities tend to be stable overtime (i.e., moderate to high test-retest reliability), the NAP task measured inhibition to visual stimuli in a way that was context-dependent [70], rendering it more efficient at predicting concurrent rather than prospective outcomes (i.e., inhibition was activated by visual stimuli that were relevant within the specific context of participants’ quality of social functioning at time 1). Ultimately, this suggests that it is challenging, based on the present data, to determine whether the relation between the predictor and outcome variables naturally weakened over time or, alternatively, our ability to measure that relation was less robust at the second time point. Future studies may require larger samples (increased power) to adequately test prospective relations between empathy, cognitive inhibition, and social functioning.

The contextual, social, and emotional cues that elicit empathy are complex. The experience of empathetic concern depends on the nature of the feelings being shared, the relationship of the individuals sharing the emotion, and the context in which the social interaction occurs. As such, the ability to understand or share in another’s emotion does not necessarily imply that one will act in a supportive or sympathetic way [18], [26], [71], [72]. In the current study, high empathy was associated with poor interpersonal functioning for individuals who displayed weak inhibition of the distracting angry faces of their partner. The present findings therefore not only highlight the complex nature of empathy, but also add to our understanding of the factors that might adversely affect empathy-related outcomes. For instance, previous studies have demonstrated high levels of empathy in individuals at risk for and suffering from depression, a disorder that is characterized by difficulties establishing and maintaining healthy interpersonal attachments [24], [73], [74]. Dysphoric and depressed individuals also tend to show inhibitory deficits on cognitive measures such as the NAP task [36], [64], [69]. Accordingly, cognitive inhibition might be a key variable in elucidating the counterintuitive notion that being vigilant to the emotional experiences of others can sometimes be detrimental to interpersonal relationships, and might be specifically relevant for understanding the social skills deficits present in depression.

As expected, individuals showed delayed responding or increased inhibition to the personally-relevant stimuli compared to the generic stimuli, supporting the use of personally-relevant stimuli in future investigations of cognitive inhibition. Importantly, the present study yielded a significant interaction between emotion type and personal-relevance, indicating that the extent to which participants demonstrated delayed responding to expressions of negative emotions was dependent on its relevance to the participant. This finding is consistent with recent neuroimaging research, which indicates that personally-relevant or familiar faces, for example, elicit different patterns of activation than those elicited by the presentation of non-familiar faces [46–50]. Moreover, as in previous studies [63], [69], we found that the NAP task was effective in eliciting inhibition, but that the inhibition was greatest when required to ignore an angry face. Both of these findings are methodologically important for future research in this area.

Some degree of caution should be employed in interpreting the results of the present study. First, only one aspect of self-regulation, namely cognitive inhibition, was assessed in this study. Future research should consider the role of other self-regulatory mechanisms (e.g., planning, activation/inhibition of behaviors) in modulating empathy levels during social interactions. Similarly, the NAP task was designed to assess individuals’ ability to prevent irrelevant emotional information from entering into working memory. As such, the present findings fail to address other inhibitory mechanisms, including individuals’ ability to withhold a pre-potent response or remove previously relevant material from working memory [34], [75]. Third, despite the widespread use of negative priming as a measure of cognitive inhibition [33], [36], [51], [63], the mechanisms by which certain stimuli elicit more or less cognitive interference (i.e., delayed responding) on the NAP task remain elusive, resulting in some debate as to whether slowed reaction times on negative priming trials actually reflect inhibitory mechanisms [76]. However, alternative explanations of the cognitive inhibition account of negative priming effects have been previously addressed and rebutted [77]. Fourth, total empathy scores, as opposed to separately considering the cognitive and affective components of empathy, were used as predictors in this study. Because the items that comprise the EQ-Short tend to tap into both cognitive and affective aspects of empathy [54], [78], assessing the unique contribution of each component on interpersonal functioning was not feasible in the current study. It is, however, possible that the importance of inhibition in modulating levels of emotional sharing during interpersonal encounters might differentially impact or be of greater relevance to certain aspects of empathy.

Sixth, our findings on personal-relevance are specific to pictures of the participants’ intimate partner and do not necessarily generalize to other types of personally-relevant stimuli (e.g., autobiographical descriptors). Moreover, during the NAP task, participants were exposed to generic pictures of emotions as portrayed by sixteen different actors compared to a single person for the personally-relevant stimuli. Therefore, participants were exposed to more repetitions of the personally-relevant faces than generic faces, which might have led participants to more readily habituate to the personally-relevant compared to the generic pictures throughout the task. Although possible, the data are not consistent with this hypothesis as stronger inhibitory effects occurred with personally-relevant pictures. Ultimately, our laboratory is among the first to explore this important research question and follow-up studies are still needed to examine alternative hypotheses. Finally, our ability to conclusively establish the directionality of the results is limited. For example, it is also plausible that empathy moderated the effect of cognitive inhibition on interpersonal functioning, or that the presence of interpersonal problems alters cognitive inhibition and ratings of empathy.

Despite these limitations, the findings of the present study expand on a growing body of literature attesting to the role of self-regulation in determining the effect of empathy on the quality of social outcomes. The current research also supports the use of personally-relevant stimuli in the study of social information processing. Ultimately, because interpersonal relationships are essential to health and well-being, knowledge of the personality and cognitive factors implicated in social functioning is crucial to the creation of prevention and treatment strategies aimed at lonely, depressed, and isolated individuals, as well as other mental health issues characterized by executive dysfunctions and social deficits (e.g., Attention Deficit Hyperactivity Disorder, Autism Spectrum Disorder) [79], [80]. For instance, these findings hold potential for the design of social-skills training [81] and attention-retraining programs [82] devised to enhance the social and emotional lives of individuals. By reducing attentional biases and promoting empathy, such programs can have a wide range of positive effects on both mental health and social functioning. Finally, given the long period of frontal lobe maturation [80], the ability to inhibit irrelevant information in the environment might have important implications with regards to the acquisition of empathy and other social skills across youth development. Namely, individual differences in frontal lobe activation may be a marker for certain social deficits and temperamental dispositions.
